# Detecting gene-by-smoking interactions in a genome-wide association study of early-onset coronary heart disease using random forests

**DOI:** 10.1186/1753-6561-3-s7-s88

**Published:** 2009-12-15

**Authors:** Matthew J Maenner, Loren C Denlinger, Asher Langton, Kristin J Meyers, Corinne D Engelman, Halcyon G Skinner

**Affiliations:** 1 Department of Population Health Sciences, University of Wisconsin School of Medicine and Public Health, 610 Walnut Street, Madison, Wisconsin 53705, USA; 2 Department of Medicine, University of Wisconsin School of Medicine and Public Health, 750 Highland Avenue, Madison, Wisconsin 53705, USA; 3 Department of Mathematics, University of Wisconsin-Madison, 480 Lincoln Drive, Madison, Wisconsin 53706, USA

## Abstract

**Background:**

Genome-wide association studies are often limited in their ability to attain their full potential due to the sheer volume of information created. We sought to use the random forest algorithm to identify single-nucleotide polymorphisms (SNPs) that may be involved in gene-by-smoking interactions related to the early-onset of coronary heart disease.

**Methods:**

Using data from the Framingham Heart Study, our analysis used a case-only design in which the outcome of interest was age of onset of early coronary heart disease.

**Results:**

Smoking status was dichotomized as ever versus never. The single SNP with the highest importance score assigned by random forests was rs2011345. This SNP was not associated with age alone in the control subjects. Using generalized estimating equations to adjust for sex and account for familial correlation, there was evidence of an interaction between rs2011345 and smoking status.

**Conclusion:**

The results of this analysis suggest that random forests may be a useful tool for identifying SNPs taking part in gene-by-environment interactions in genome-wide association studies.

## Background

The prospects and promises of the completed Human Genome Project include improvement in the development of novel therapeutics, intervention strategies for personalized treatment of common diseases, and new diagnostic methods for both rare and common diseases. While completion of the project took years, recent technological advances have reduced costs such that genome-wide association studies (GWAS) are a popular design for genetic epidemiological studies. However, the computational matrix remains daunting, often with genotypes from several hundred thousand SNPs collected on thousands of individuals.

Coronary heart disease (CHD) is a common disorder with multiple risk factors and a recognized likelihood of significant gene-by-environment interactions. As an example, a candidate gene approach has shown that the hazard ratio of composite cardiovascular disease is elevated preferentially in smokers having the APOE ε4 allele, with a significant interaction between genotype and smoking status [1,2].

Though GWAS may include hundreds of thousands of single-nucleotide polymorphisms (SNPs), current computational and practical limitations often prevent researchers from fully exploiting the genetic information available. The rate at which genetic data are generated greatly outpaces our ability to fully analyze them. Many studies are faced with difficult choices to make the analyses feasible. Even though there is evidence that many diseases do not follow the single-gene mendelian inheritance model, genetic analyses frequently cannot account for all possible interactions due to limited computational resources.

For these reasons, machine learning has become increasingly popular with large genetic datasets. Machine learning involves iterative building of predictive models from complex datasets, and has the potential to resolve these genome-wide computational challenges. The random forest (RF) algorithm, a type of machine learning, can be used to reduce the data and identify potential gene-by-environment interactions. This approach is an appealing alternative to other analytical methods for GWAS that typically base selection of "significance" entirely on *p*-values. As a part of Genetic Analysis Workshop 16 (GAW16), this paper explores the ability of RF algorithms to identify SNPs that may be involved in gene-by-smoking interactions related to our outcome of interest, age of early onset CHD.

## Methods

### Dataset

The Framingham Heart Study (FHS) GWAS data (GAW16 Problem 2) was used in this analysis. The data includes genotype, phenotype, environmental exposure status, and family structure information on 7,130 participants from three enrolment periods with cohort follow-up of over 100,000 person-years (estimated). Local institutional review board approval was obtained.

### Study design

We used age at incident of CHD or censoring as the outcome variable. Hard CHD was classified as recognized myocardial infarction diagnosed through an electrocardiogram or enzymes, coronary insufficiency, or death attributed to CHD. The Third Generation of FHS was excluded from analyses because there was no follow-up information available beyond enrollment, there were only 26 cases of CHD, and most of these were considered previously existing due to minimal information at the enrollment visit regarding the CHD incident date.

Dense genotyping of approximately 550,000 SNPs across 22 autosomal chromosomes for each FHS subject was performed by Affymetrix using the 250 k Sty, 250 k Nsp, and the supplemental 50 k platforms (GeneChip Human Mapping 500 k Array Set and the 50 k Human Gene Focused Panel). Quality control of the genotype data was performed with PLINK v1.0.3 [3,4]. SNPs were eliminated if the minor allele frequency was <5% (111,290 SNPs), if >5% of the data was missing for a single SNP (31,975 SNPs), or if the genotype frequencies deviated significantly from Hardy-Weinberg equilibrium (*p *< 0.001; 12,622 SNPs). A total of 355,649 SNPs met all quality-control criteria. Additionally, 29 individuals missing data for >5% of the SNPs were removed.

Phenotype and exposure information were tabulated with SAS v9.1.3 (SAS Institute Inc, Cary, NC) and a categorical ever/never smoking status variable was created using the most recent data from a visit before incident CHD. To account for potential period effects, we estimated the decade of birth for each participant and created a categorical variable for each decade. While the RF algorithm cannot account for pedigree explicitly, we created binary indicators for each family ID to include as covariates. We used perl scripts for data management, creating a singe flat file that merged genotype, pedigree, phenotype, and exposure data.

### RF analysis

The RF algorithm is a machine-learning classifier made up of many decision trees and has qualities well suited for genome-wide, gene-by-environment and gene-by-gene interaction studies [5]. Each tree is built from a training set constructed by sampling a number of cases with replacement at random from the data. At a given node (i.e., decision point), the algorithm finds the variable that partitions the remaining cases into two subsets, *L *and *R*, so as to maximize the heuristic best-split criterion:

The procedure continues recursively, so that the subsets *L *and *R *are partitioned by left and right child nodes, respectively, and terminates when the partitions are sufficiently small. The resulting terminal nodes are assigned values equal to the mean of the outcome (i.e., age) in the corresponding partitions. This process is repeated to produce many decision trees (a forest) whose predicted outcomes are combined into a single value by taking a majority vote (for categorical outcomes) or finding the mean value (for numerical outcomes). The data that were excluded from the training set form the *testing set*, which is used to gauge the accuracy of a tree. To measure the importance of a particular variable on a given tree, the values of that variable are randomly permuted among the data in the testing set and the predictions are recomputed. The difference between the two predictions provides a measure of variable importance, with important variables showing a larger discrepancy between the predictions.

The RF was run using a derivative of the publicly available RF regression code [6], rewritten in the C programming language. The new program follows the same approach described by Breiman, but adds memory management optimizations that make it feasible to perform a RF analysis using several hundred thousand input variables [7]. Each tree was built using 224 cases sampled *with replacement *from the total pool of 224 cases via bootstrap aggregating, a process that allows for simulation of larger data sets and minimizes overfitting that can occur in traditional training/validation set design. Following Breiman's recommendations, one-third of the input variables (includes all SNPs, covariates, and binary family ID variables) were randomly chosen for consideration at each node in a decision tree, and the variable from this subset that produced the best (heuristic) split was found. The tree nodes were recursively split until at most five cases remained in each terminal node.

The inclusion in the data set of multiple members from a single family could result in a SNP mistakenly being reported as important. To guard against this possibility, the importance of the family ID variable was also measured. If a SNP appeared to be important solely due to similarity between family members, then the family ID variable would also be reported as important.

SNPs identified as important by RF for determining the outcome (age of CHD onset) were run in the controls to determine whether the association was age-related. Smoking status was treated as a simple covariate in the RF model. The interaction between the SNP, smoking status, and CHD was then determined using generalized estimating equations (GEE) via PROC GENMOD in SAS to account for family structure and adjust for sex. The number of alleles was treated as a class variable, therefore assuming no genetic model.

## Results

**Figure 1:**
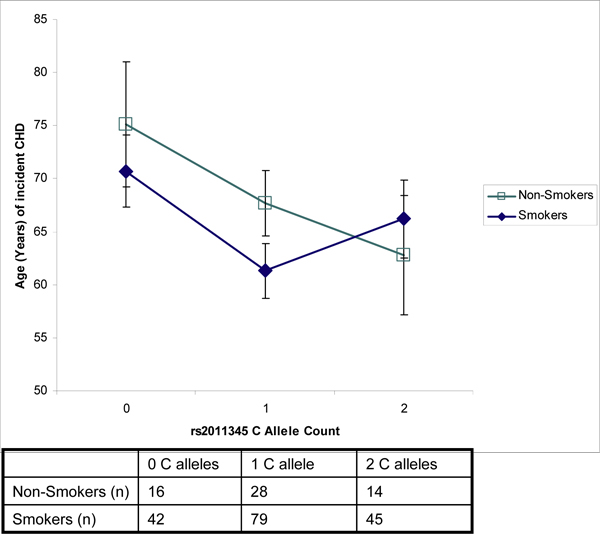
**Mean age of CHD event among non-smokers and smokers by rs2011345 C allele count**. Age of CHD onset adjusted for sex and family membership. Vertical bars represent 95% confidence intervals.

From the combined Original Cohort and Offspring Cohort of FHS, there were 224 incident CHD cases and 2,909 controls with complete data meeting the quality control criteria discussed above; 166 of the cases were classified as ever-smoked at the visit before the incident event, and of the 224 cases, there were 167 unique family IDs (i.e., there was only one individual in the family) and only 38 cases were a first-degree relative of one of the other cases. Among controls, 1,530 were classified as "ever smoked" at the last visit with available data. The RF algorithm generates a variable importance score for each variable included in the analysis. After ranking the covariate importance score in each of four runs of RF using 500 trees each, there was one SNP (rs2011345) that ranked as the most important SNP and within the top 10 of all ranked covariates in three of the four runs (Table 1). The GEE results for the selected SNP showed a main effect for both the SNP and smoking status, as well as evidence of an interaction. The interaction term was significant by a -2 log likelihood-ratio test comparing the full and reduced models (*p*-value = 0.002). In the 0-allele group, non-smokers had a mean age of incident CHD of 75.1 yr compared to 70.7 yr among smokers (Figure 1). This was not statistically significant (Wald *p*-value of 0.19). There was a statistically significant difference between smokers and non-smokers in the 1-allele group, where non-smokers had a higher mean age of incident CHD compared to smokers (67.7 vs. 61.3 yr), with a Wald *p*-value of 0.003. The 2-allele group did not differ statistically (Wald *p*-value 0.30), but the direction of the relationship differed such that smokers had a slightly higher mean age of incident CHD (62.8 vs. 66.2 yr). Similar results were obtained using *t*-tests when ignoring family membership and when different correlation matrices were specified in the GEE procedure.

**Table 1:** Top 10 covariates returned by four RF runs with 500 trees

	Run 1		Run 2		Run 3		Run 4^a^	
				
Rank	Covariate	Importance	Covariate	Importance	Covariate	Importance	Covariate	Importance
1	Birth decade 1900	16.3077	Birth decade 1900	20.2087	Birth decade 1900	17.6408	Birth decade 1900	19.6011
2	Birth decade 1930	8.8219	Birth decade 1930	8.2609	Birth decade 1930	11.7332	Birth decade 1930	10.2538
3	Smoking	1.7807	Smoking	2.5351	Birth decade 1920	1.7106	Smoking	2.8397
4	Birth decade 1920	1.1131	Birth decade 1920	0.6642	Smoking	1.6917	Birth decade 1920	1.6002
5	Sex	0.9290	rs2011345_C	0.4265	Sex	0.8159	Sex	0.4328
6	rs2011345_C	0.4912	rs32732_G	0.3341	rs3866685_A	0.2945	rs2566762_T	0.2865
7	rs9349061_T	0.4427	rs17456025_A	0.3095	rs2011345_C	0.2915	rs549582_T	0.2757
	rs732998_T	0.2739	rs2396500_G	0.2993	rs4849404_C	0.2714	rs963274_T	0.2424
9	rs949753_G	0.2719	Sex	0.2935	Birth decade 1910	0.2694	rs4490198_G	0.2059
10	rs1686567_A	0.2664	rs6565249_T	0.2538	rs12052316_A	0.2594	rs17456025_A	0.2047

^a^rs2011345 was ranked 30^th^, with an importance score of 0.1268

To determine whether the identified SNP was generally associated with age rather than age at CHD onset, the same analysis of rs2011345 was run among the control group with age at last contact as the outcome, and there were no statistically significant main effects or interactions present. This suggests that the association found among the cases is not due only to the age or survival of the participants.

## Conclusion

Given the computational challenges of gene-by-environment interaction analysis in the setting of GWAS, we sought to optimize a publicly available machine learning algorithm to minimize hardware limitations. The optimizations were mostly related to memory use. Comparing execution speed using datasets smaller than the full FHS GWAS data, the optimized code ran in about 1/30^th ^of the time needed to execute the original code. Future analyses could include formal testing of the computational efficiency with increasing dataset sizes.

The optimized RF algorithm identified smoking status and the rs2011345 SNP as important classifiers in RF. Subsequent regression models confirmed that the SNP and smoking status had significant main effects with the outcome, and also a significant interaction. While RF deemed this SNP important, it may have been overlooked had the analysis been based exclusively on regression and *p*-values. Out of the 355,649 SNPs tested in standard tests of association using linear regression, rs2011345 ranked as the 2,111^th ^smallest *p*-value (*p *= 0.006) in a sex-adjusted model, and only the 29,776^th ^smallest *p*-value (*p *= 0.079) for the sex-adjusted SNP-by-smoking status interaction model in PLINK (treating allele count as a linear variable). Other important covariates identified by the RF runs included sex, smoking status, and decade of birth--the latter picking up a potential survival bias where older individuals included in the study are more likely to have a later age of onset.

Whereas the relative magnitude of this interaction compared with all other potential interactions is presently untested, the following correlative information is of interest. SNP rs2011345 is approximately 11 kb from the 3' end of the flavin-containing monooxygenase 4 gene (*FMO4*, Map Viewer build 36.3). This region appears to be in linkage disequilibrium with the last two exons of *FMO4 *in the CEU cohort from the HapMap project (build 36). This gene is part of a family of enzymes involved in the metabolism of nicotine and other tobacco-related products. Additionally, this region (1q24) has been linked to essential hypertension [8]. Thus, the highest ranked candidate of this optimized RF algorithm has at least minimal biological plausibility worth exploring in future studies of CHD.

Our study has a few limitations. First, sample size limitations should be considered; while the interaction between rs2011345 and smoking was statistically significant, the study contained only 224 incident cases of CHD. Second, the interpretation of results is also limited by a relatively crude measure of smoking exposure. The available information on smoking behavior only provided a cross-sectional glimpse of the subject's smoking habits and, in some cases, incident CHD occurred a decade or more after the last known smoking status. Furthermore, the RF algorithm cannot account for familial correlation using traditional approaches. However, we were able to confirm the RF findings using a GEE model that accounts for familial correlation. While the 224 cases in this analysis did not have a large degree of relatedness, it could be an issue in the larger FHS population.

Future work addressing practical issues of RF may enhance its attractiveness as an analysis tool for GWAS. We used runs of 500 trees in this analysis and found the top covariates in the output to be more stable than in runs of 200 trees. Runs of 1,000 and 1,500 trees did not produce markedly different results. Another important problem is reconciling the different SNPs identified by RF with traditional *p*-value based methods. Nonetheless, it appears as though RF can provide an alternative to traditional regression techniques to reduce the high-dimensional data space of GWAS searching for gene-by-environment interactions.

## List of abbreviations used

CHD: Coronary heart disease; FHS: Framingham Heart Study; GAW: Genetic Analysis Workshop; GEE: Generalized estimating equations; GWAS: Genome-wide association studies; RF: Random forests; SNP: Single-nucleotide polymorphism.

## Competing interests

The authors declare that they have no competing interests.

## Authors' contributions

MJM contributed to the study design, data manipulation, reduction, non-RF statistical analyses, and drafted sections of the manuscript. LCD contributed to the conception and development of the study design, review of the literature, and drafted sections of the manuscript. AL programmed and ran the RF analysis and drafted sections of the manuscript. KJM provided statistical and methodological consultation. CDE and HGS supervised the work on this paper as part of the Genetic Analysis Workshop and provided assistance with the data manipulation. All authors participated in the review, discussion, and critiquing of the drafts of this paper.
